# Urinary bladder fistula following laparoscopic inguinal hernioplasty: a case report

**DOI:** 10.1186/s12893-021-01183-6

**Published:** 2021-04-07

**Authors:** Ken Hagiwara, Shigeoki Hayashi, Takeki Suzuki, Keio Song, Tadatoshi Takayama

**Affiliations:** 1 Department of Digestive Surgery, Nihon University School of Medicine, 1-6 Kandasurugadai, Chiyoda-ku, Tokyo, 101-8309 Japan; 2 Department of Surgery, Toride Medical Association Hospital, Ibaraki, Japan

**Keywords:** Cystoscopy, Dysuria, Inguinal hernia, Surgical mesh, Trans-abdominal preperitoneal repair, Fistula, Mesh erosion, Urinary bladder

## Abstract

**Background:**

Fistula formation due to mesh erosion into hollow viscera, such as the urinary bladder, is uncommon. To date, there have been no reports of fistula formation into the urinary bladder without evidence of mesh erosion after hernioplasty; herein, we report one such rare case, in which the clinical symptoms improved without any surgical intervention.

**Case presentation:**

A 73-year-old man underwent a trans-abdominal preperitoneal repair for bilateral direct inguinal hernia. One month later, the patient experienced a painful induration in the right inguinal region, and computed tomography revealed fluid collection in this region. A culture of the aspirated fluid yielded no bacteria. Seven months later, he experienced another episode of painful induration in the same region. However, blood examination revealed a normal white blood cell count and C-reactive protein level. Moreover, no organisms were detected by aspirated fluid culture. Although the painful induration subsided after aspiration of the fluid collection, he developed gross hematuria and dysuria a month later. Cystoscopy revealed a fistula in the right wall of the urinary bladder that discharged a purulent fluid. Culture of the fluid revealed no bacteria, and there was no evidence of mesh erosion. Hematuria improved without therapeutic or surgical intervention. The patient’s clinical symptoms improved without mesh removal. Moreover, cystoscopy revealed that the fistula was scarred 12 months after the initial appearance of urinary symptoms. No further complications were observed during a 42-month follow-up period.

**Conclusions:**

We report a rare case of a fistula in the urinary bladder without evidence of mesh erosion after laparoscopic hernioplasty. The patient’s condition improved without mesh removal. Fluid collection due to foreign body reaction to meshes can cause fistula formation in the urinary bladder without direct mesh contact.

## Background

Tension-free hernia repair with prosthetic mesh is associated with lower recurrence rates and has become the standard treatment for inguinal hernia [1, 2]. Fistula formation due to mesh erosion into hollow viscera, such as the urinary bladder, is an uncommon adverse event [3–8]. Although rare, urinary bladder fistulae due to various inflammatory diseases and unrelated to meshes have been reported occasionally [9, 10]. However, to date, no urinary bladder fistula has ever been reported after hernia repair without evidence of mesh erosion. Herein, we report a rare case of a fistula in the urinary bladder, in which the patient’s clinical symptoms improved without mesh removal.

## Case presentation

**Fig. 1:**
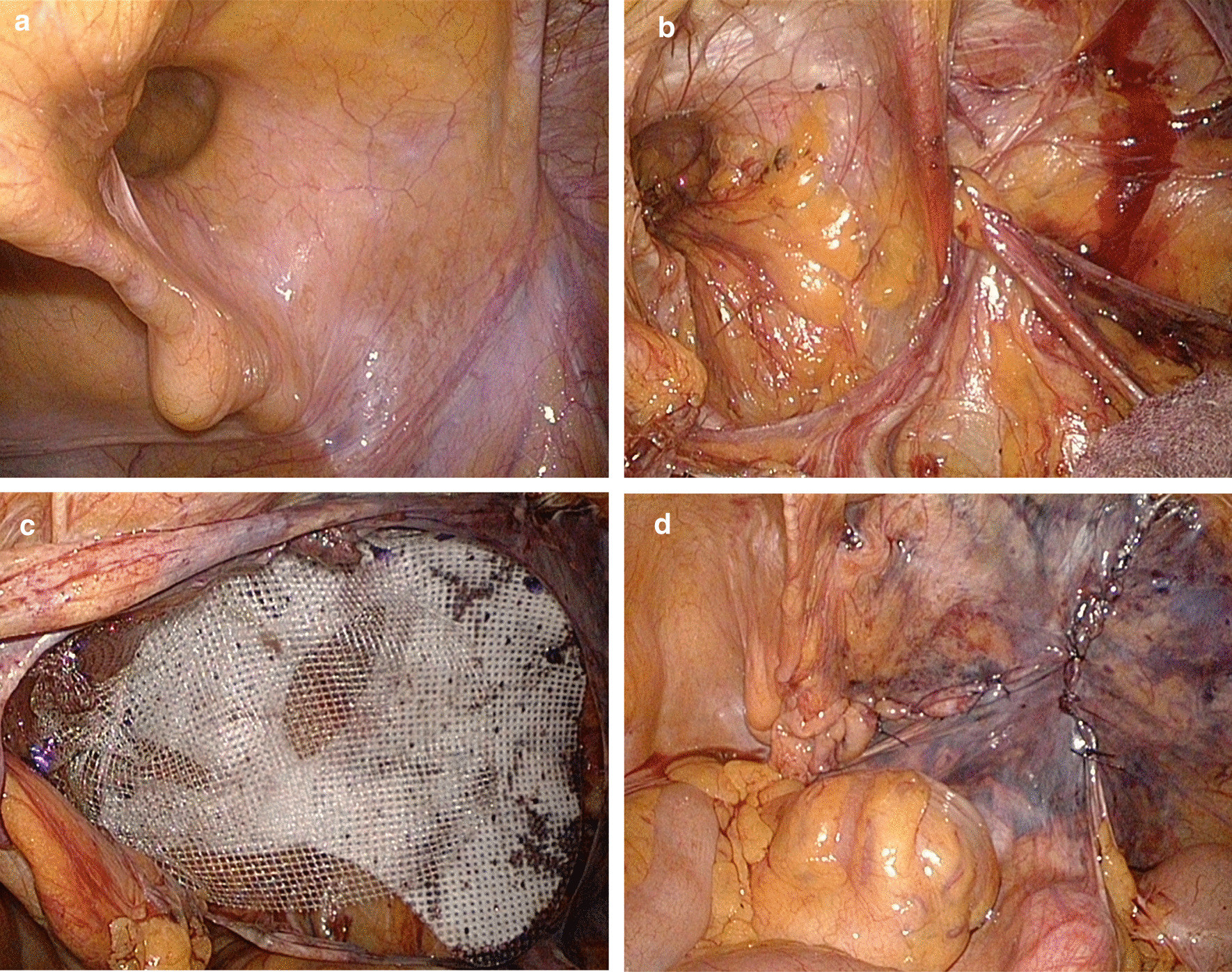
Intraoperative findings. **a** laparoscopic view before hernioplasty, **b** laparoscopic view after preperitoneal dissection, **c** mesh fixation, **d** peritoneal closure

**Fig. 2:**
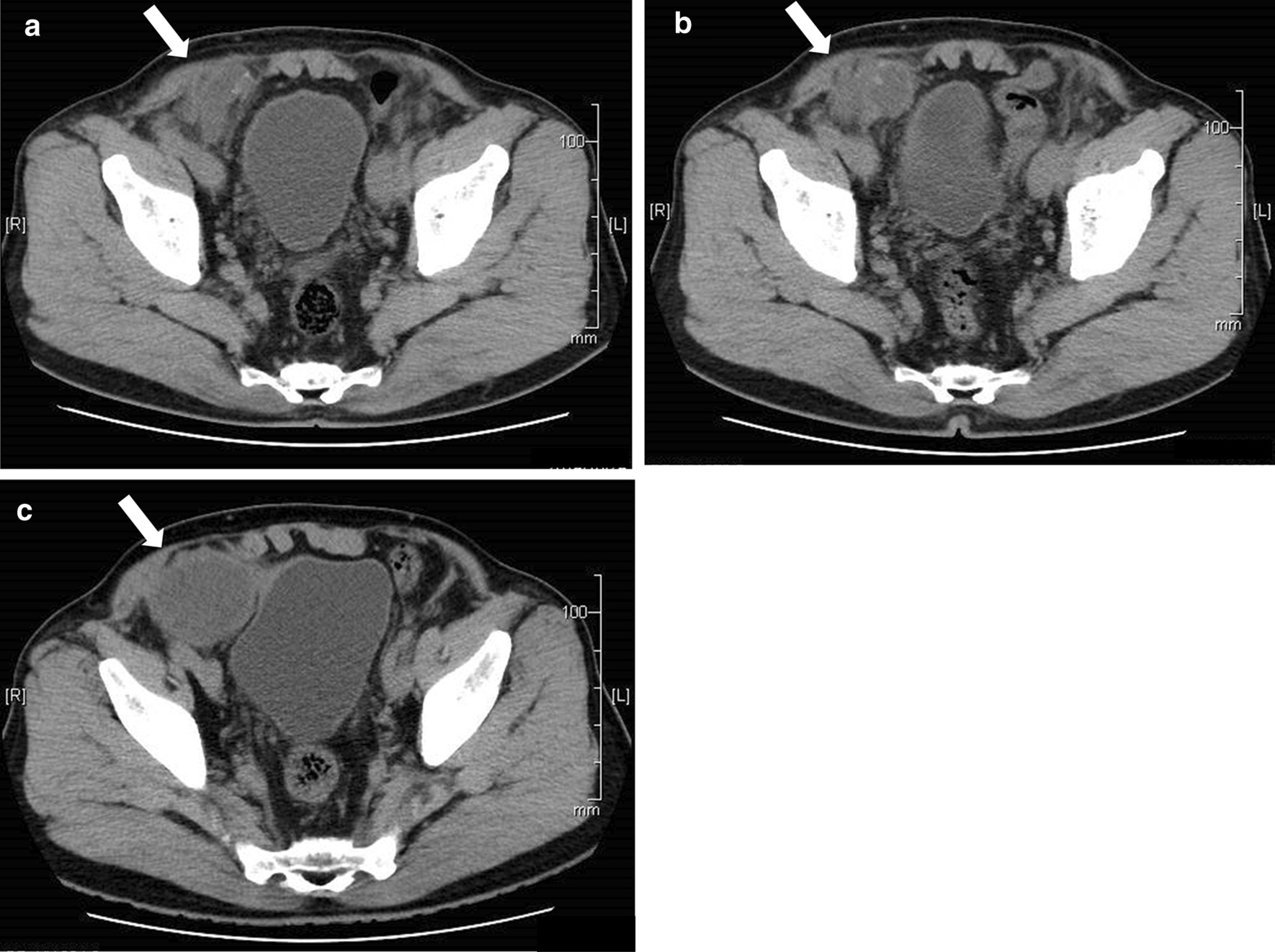
Computed tomography shows fluid collection in the right inguinal lesion (arrow, fluid collection) **a** 1 month after surgery, **b** 3 months after surgery **c** 10 months after surgery

A 73-year-old man was referred to our hospital with bilateral direct inguinal hernia. A trans-abdominal preperitoneal (TAPP) repair was performed using a polypropylene mesh. Operative findings revealed that the hernia content did not include the urinary bladder and that the mesh, which was fixed in the preperitoneal space, was thoroughly dissected. Furthermore, the urinary bladder was not exposed, and the mesh was not in direct contact with it. This mesh was fixed to the lateral border of the rectus abdominis muscle, transverse abdominal fascia, Cooper's ligament, and pubic tuberosity using an absorbable fixation device. The peritoneum was closed with continuous sutures using a 3–0 absorbable braid thread. There were no notable complications during peritoneal closure. (Fig. 1) The postoperative course was uneventful. One month later, the patient noticed a painful induration in the right inguinal region; computed tomography (CT) revealed fluid collection in this region (Fig. 2a). The patient had no fever. Blood tests revealed a normal white blood cell (WBC) count and C-reactive protein (CRP) level. Three months after surgery, CT revealed an increase in the fluid collection (Fig. 2b). CT scans taken both 1 and 3 months after the surgery also revealed an interval between the fluid collection and the bladder, indicating no obvious continuity between them. Serial aspirations of the fluid collection were performed, and no organism was identified on aspirate culture. The fluid had a pus-like appearance; it was neither bloody nor serous, yellowish, and clear that would have otherwise indicated a hematoma or a lymphocele, respectively. Suspecting mesh-related infections, we suggested a surgical intervention; however, as the painful induration subsided following fluid aspiration, the patient wished to undergo a follow-up without mesh removal.

**Fig. 3:**
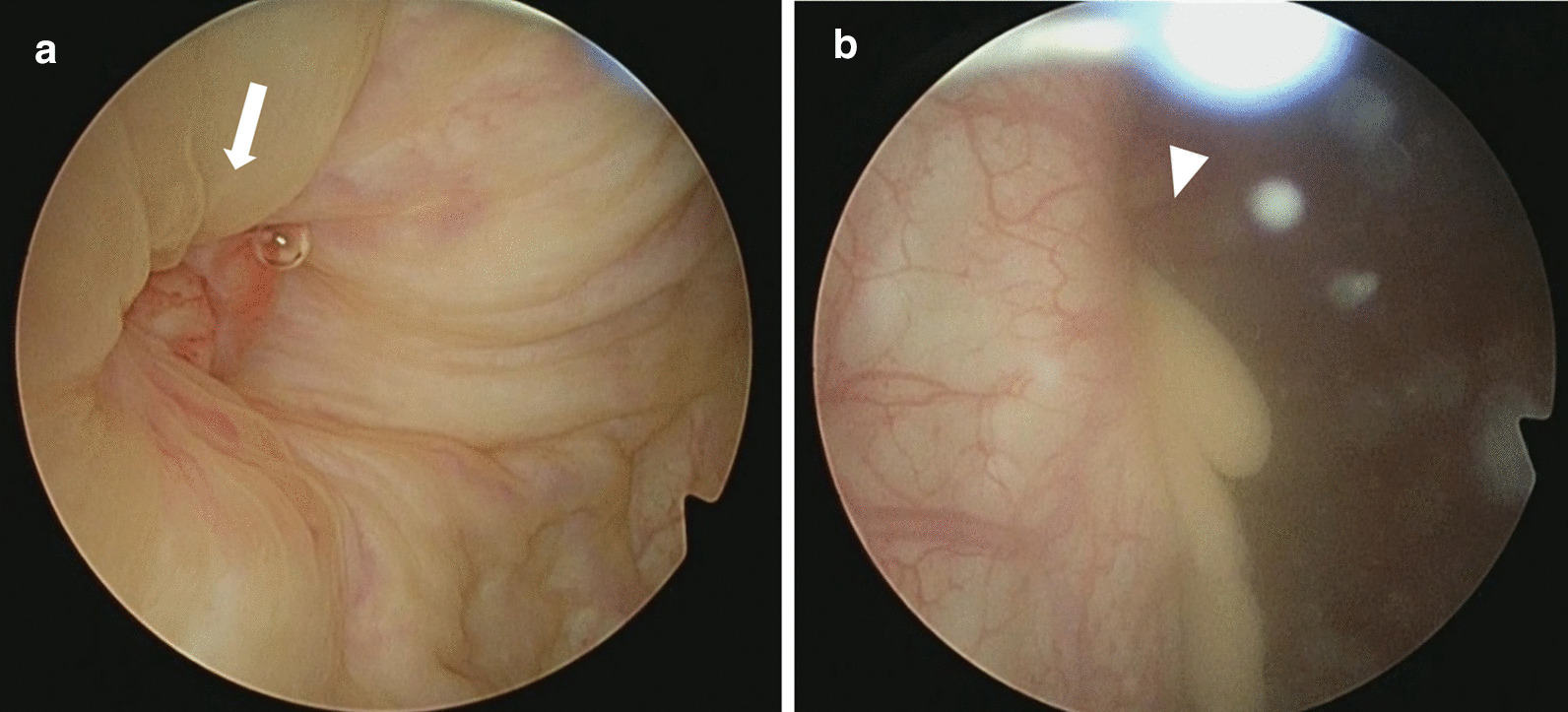
Cystoscopy shows granulation (**a**, arrow) and pus drainage (**b**, arrowhead) on the right side of the bladder

**Fig. 4:**
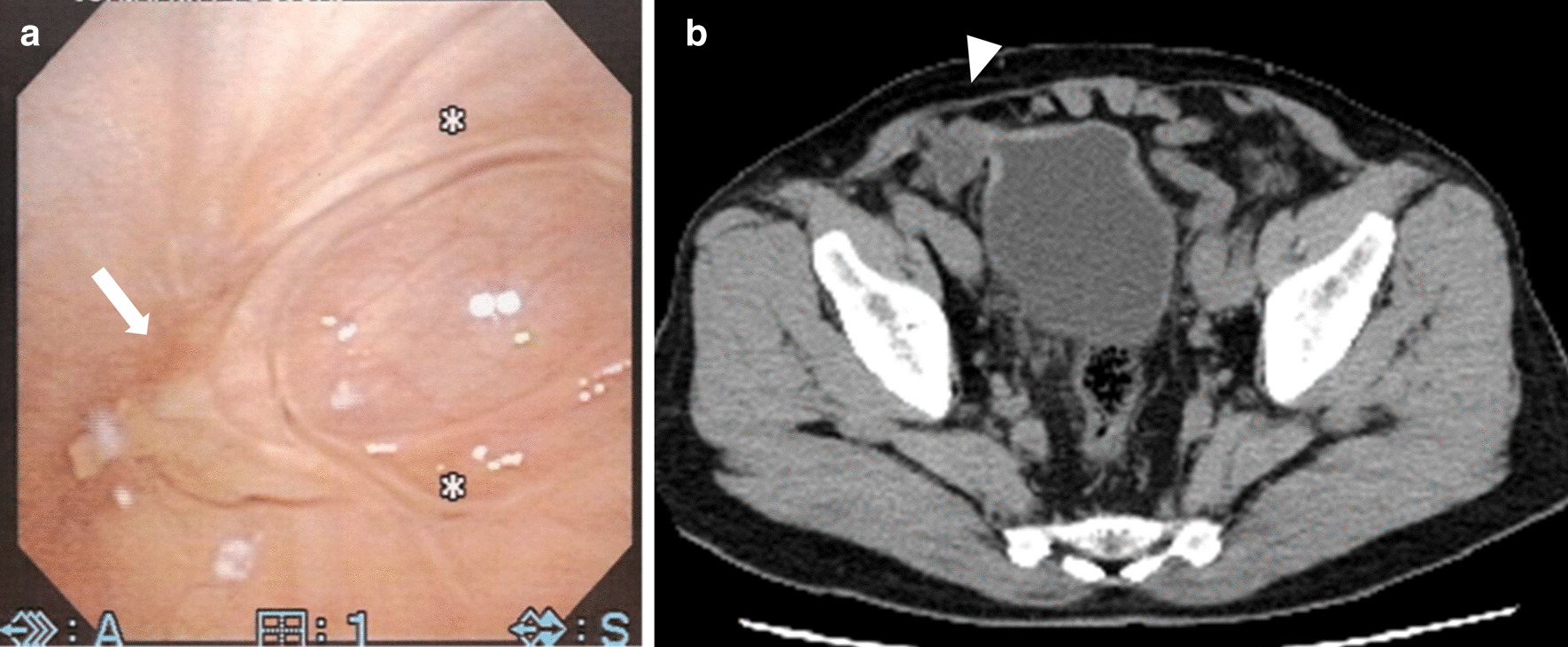
**a** Cystoscopy 5 months after the second cystoscopy shows a healed fistula (arrow). **b** Computed tomography scan showing reduced fluid collection (arrowhead)

Seven months after the aspiration (10 months after the surgery), he experienced another episode of painful induration in the right inguinal region. While blood examination continued to show normal WBC and CRP levels, CT revealed fluid collection in the right inguinal region (Fig. 2c). We performed an aspiration, and the aspirate was purulent in appearance as before, being neither bloody nor lymphatic. No organism was found by aspirated fluid culture. Urinalysis was negative for all items including occult blood, WBCs, and nitrite. A urine sedimentation test was also negative for all parameters including red blood cells, WBCs, transitional epithelium, and bacteria. Although the painful induration subsided after aspiration of the fluid collection, he developed gross hematuria and dysuria a month later and visited a urologist. Cystoscopy revealed granulation on the right urinary bladder wall, and a biopsy revealed a dysplastic urothelium. Therefore, the patient was referred to our hospital. Despite improvement in dysuria without any intervention, gross hematuria persisted. Although we suggested mesh removal, the patient refused surgery as his urinary symptoms were improving. Considering the possibility of a malignant tumor, the patient underwent a second cystoscopy that indicated a fistula in the right urinary bladder wall that was discharging purulent fluids (Fig. 3). These purulent fluids were similar to what we had aspirated previously. Furthermore, the fluid drained profusely upon compression of the right inguinal region. Histological examination of the biopsy specimen indicated granulation, but no malignancy. Culture testing revealed no bacteria, and there was no evidence of mesh erosion. Hematuria improved without therapeutic or surgical intervention 3 months after the second cystoscopy. Furthermore, 5 months after the second cystoscopy, CT revealed a reduction in fluid collection, and cystoscopy indicated that the fistula was scarred (Fig. 4). No further complications such as hematuria, dysuria, or mesh erosion in the urinary bladder were observed during a 42-month follow-up period.

## Discussion and conclusions

We encountered a rare case of fistula formation in the urinary bladder due to fluid collection around the mesh after TAPP repair. There are two important clinical observations in this case. First, although it is assumed that mesh contact is the primary reason for fistula formation, fluid collection could also cause fistula formation in the urinary bladder without direct mesh contact. Second, the patient’s condition improved without mesh removal, suggesting that surgical intervention may not be necessary in all cases.

Fistula formation in the urinary bladder due to mesh erosion has occasionally been reported as an uncommon mesh-related complication [11]. However, to the best of our knowledge, this is the first report of fistula formation in the urinary bladder without mesh erosion. We believe that identifying such cases where fistula formation is not associated with mesh erosion is crucial, because fistula formation in the urinary bladder can occur without direct contact between the mesh and the bladder. Additionally, these cases may not necessarily require invasive surgical interventions, such as mesh removal, to improve the symptoms.

Mesh erosion is assumed to occur due to technical factors, such as inadequate detachment of the peritoneum and incorrect placement of the mesh. Direct contact between the mesh and the urinary bladder causes fistula formation, resulting in mesh erosion [8]. Therefore, it is important to cover the myopectineal orifice by sufficiently advancing and securely fixing the mesh, while ensuring that it is not in direct contact with the urinary bladder after securing adequate space in the preperitoneal cavity. In this case, we hypothesized that fluid collection around the mesh, rather than a direct contact with the mesh itself, caused fistula formation into the urinary bladder, leading to fluid drainage. We deduced this hypothesis for three reasons. First, we ensured that the TAPP procedure was completed without any complications. Furthermore, after sufficient dissection, the mesh was fixed without any direct contact with the urinary bladder. Second, although previous reports showed that the mesh was exposed in all cases, cystoscopies in the present case revealed no exposure of the mesh into the urinary bladder. Third, we observed fluid collection around the mesh several times, following which aspirations were performed and the symptoms improved. However, he experienced pain on urination and hematuria. While CT initially showed no findings of an overt fistula between the bladder and the fluid collection, it eventually revealed a high-density area that was suggestive of fistula formation. Furthermore, urinalysis one month prior to the visit to the urologist revealed no obvious findings. These suggest that the fistula was first caused by fluid collection, and later developed into a bladder fistula. In previous reports, direct contact between the mesh and the bladder initially exposed the mesh to the urinary bladder, resulting in mesh-related infections. The present case had a completely different clinical course than the previous cases.

Previous reports have shown that total or partial mesh removal was necessary to improve symptoms in cases involving mesh erosion into the urinary bladder [11]. Moreover, some cases required partial cystectomy [5, 6]. In the present case, mesh removal was considered at the onset of the symptoms; however, the patient did not wish to undergo surgery and the symptoms improved without mesh removal. Additionally, it was reported that meshes should be removed once they become infected in order to control an infection [3–8, 10–13]. In this case, blood examination and culture of both, the fluid aspirate and urine, revealed no bacterial infection. Local irritation could lead to localized tissue inflammation with little or no systemic components. In a majority of these cases, blood-based investigations would not yield any significant results. These results suggest that fluid collection could be due to a foreign body reaction to the mesh and not due to infection. Conservative treatment is undertaken in the absence of mesh-related infections following fistula formation [12]. Our findings suggest that in some cases of fistula formation, in the absence of an infection, symptoms could improve even without mesh removal. Therefore, unnecessary over-invasive interventions should be avoided. Previous studies have indicated that mesh erosion was frequently observed in cases where more than 10 years had passed after the surgery [6, 7]. Moreover, there have been reports of cases where mesh removal was required even though fistula formation was due to a foreign body reaction [13]. In our case, cystoscopy showed no mesh erosion into the bladder during the 42-month follow-up period. However, a longer follow-up period is necessary to account for delayed mesh exposure. Furthermore, although several cystoscopies confirmed the absence of mesh erosion in the present case, laparoscopic assessment would have helped determine if the mesh had eroded the bladder or not. In this case, although fluid collection and a fistula to the bladder were diagnosed based on the clinical course and cystoscopy findings, a cystography should be considered when the diagnosis is difficult.

In conclusion, we experienced an extremely rare case of fistula formation in the urinary bladder due to fluid accumulation around the mesh after TAPP repair. In this case, the symptoms showed long-term improvement without mesh removal. Further investigation on the mechanism of fistula formation without mesh erosion and its appropriate treatment is warranted.

## Data Availability

Data sharing is not applicable to this article as no datasets were generated or analyzed during the current study.

## Abbreviations

TAPP: Trans-abdominal preperitoneal
WBC: White blood count
CRP: C-reactive protein
